# Physical fitness percentiles of Polish children aged 4–7 years

**DOI:** 10.1038/s41598-021-86903-x

**Published:** 2021-04-01

**Authors:** Karolina H. Przednowek, Marta Niewczas, Łukasz Wójcik, Wojciech Paśko, Janusz Iskra, Krzysztof Przednowek

**Affiliations:** 1 Institute of Physical Culture Sciences, Medical College of Rzeszów University, Rzeszów University, 35-959 Rzeszów, Poland; 2 Lower Silesian Regional Association of Traditional Karate, 50–529 Wroclaw, Poland; 3 Faculty of Physical Education and Physiotherapy, Opole University of Technology, 45-758 Opole, Poland

**Keywords:** Health care, Public health, Population screening

## Abstract

The purpose of this study was to report sex- and age-specific physical fitness level in Polish children aged 4 to 7. 11.709 children participated in the study, including 5.684 girls and 6.025 boys aged 4 to 7 who attended kindergarten institutions throughout Poland. Physical fitness was assessed using four tests developed by Sekita including shuttle run 4 × 5 m with moving the block, standing long jump, throwing 1 kg medicine ball with two hands above the head and 20 m run. Percentile charts were developed separately for males and females using the LMS method. Boys showed higher physical fitness values than girls. In addition, an increase in the level of physical fitness was observed along with the age of the subjects. The developed reference values by age and sex in the field of physical fitness can be used for diagnostic purposes and assessing the level of physical fitness of preschool children. In addition, they can be helpful for healthcare professionals, parents and teachers to develop children’s motor activation programs and monitor their physical fitness.

## Introduction

Physical fitness is a fundamental factor in proper development during childhood and adolescence^1–3^. It can, however, be assessed through various components, such as body composition, cardiopulmonary fitness, musculoskeletal fitness, motor fitness and flexibility. Currently, more than 15 tests are available around the world to assess the fitness level of children and adults. The most common of them are Eurofit, FitnessGram and Alpha-fit^3^. Cvejić assessed physical fitness with the Alpha-fit test, which included 4 trials: handgrip strength test, standing long jump, 20 m shuttle run test and 4 × 10 m shuttle run test^2^. Valentini used the Test of Gross Motor Development (TGMD-2) for motor assessment, which consisted of such components as run, hop, horizontal jump, leap or overhand throw^4^. Early assessment of preschool children is particularly important for monitoring the changes in their motor development, identifying developmental delays or deficiencies and for assistance in creating physical exercise programs^4^. Although the symptoms of many diseases appear in adulthood, the onset of the disease usually occurs in childhood^5^. Studies indicate that a higher level of physical fitness in childhood was associated with more positive health outcomes regarding the current and future risk of many diseases, such as obesity or cardiovascular diseases^6,7^. According to Ortega et al. high levels of cardiovascular fitness and muscular fitness improve the functioning of the cardiovascular system in young people^6^. Other report have shown that very low levels of muscle strength during puberty are associated with a higher risk of premature death from any cause before the age of 55, as well as due to cardiovascular disease^8,9^. Studies also show that preschool children (5 years old) with high levels of physical fitness were less obese and more concentrated. Such conclusions were reached by Lang et al. assessing cardiorespiratory fitness with the 20 m shuttle run test^10^. Qi et al. observed that after a 24-week training session, the group of studied children improved their motor skills (30-s speed tapping and throw a tennis ball) and hand grip strength, compared to the control group^11^. Leading an active lifestyle at this stage of life has also a positive effect on the child’s physical, cognitive and mental health^6,12–14^. Cantell et al. emphasize that a high level of physical fitness (ball catching and throwing, jump and clap, static and dynamic balance, foot and finger tapping at this age has a positive effect on the learning process and school achievements^15^. In addition, early years of life facilitate the acquisition of basic motor skills, which are associated with creating more complex and specialized movements^16^.

Analysis of the physical condition of Polish children in the years 1999–2009 showed a deterioration in the physical fitness of children and adolescents aged 6–19^17^. Studies by Wolański and Dobosz have also demonstrated that negative tendencies in motor development also appear in younger children^18^. The recorded regress concerned 6 out of 9 motor tests and the largest was expressed in the flexibility, endurance and strength. Other reports have proven that, generally, the level of physical activity of children during the preschool period is systematically decreasing^19^. Many studies have shown that young children spend most of the day sitting and less than 5 percent of the day on moderate-to-vigorous physical activities (MVPA)^20–22^. Data collected by the American Obesity Association (2004) and U.S. Department of Health and Human Services (2004) suggest that children are moving less and adopting a more sedentary lifestyle^23^.

In the European countries studies have show the reference values for physical fitness of children and adolescents^24–27^. However, due to the fact that the level of physical fitness is determined by biological and environmental factors, which are different in individual countries, it is impossible to compare the physical fitness of Polish children with the norms in other European countries. In Poland, percentile charts for physical fitness standards have been developed for children and adolescents aged 7–19^28^. No studies have been conducted that would provide reference values for physical fitness in Polish children under 6 years of age (preschool). Therefore the main purpose of this study was to provide reference percentile charts for the following parameters of physical fitness. The research used the Wroclaw Physical Fitness Test for preschool children developed by Sekita. The reason for choosing this method was its availability and simplicity in carrying out all the trials. This test covers the most important motor skills: strength (throwing a medicine ball weighing 1 kg), speed (20 m run), agility (shuttle run 4 × 5 m with moving the block) and power (standing long jump). Choosing a test to assess children’s physical fitness, the authors also found that these tests were adapted to the level of motor abilities of preschool children.

## Material and methods

### Sample

The research was carried out in 2016-2019 as part of the Ministerial project “Little Wonderful”. The project involved measuring the level of children’s physical activity only. 11.709 children (5.684 girls and 6.025 boys) aged 4 to 7 participated. Decimal age was obtained from each child, and age groups were based on a complete year (i.e.. 5.00 and 5.99 etc.). The most numerous group were children aged 5 (4231 people) and 6 (4359 people). The smallest group were 7-year-old children (1.194 people). More detailed data are included in Table 1. The children attended public preschool institutions throughout Poland. Sick, intellectually or physically disabled children were excluded from the study. Guardians of all the children expressed their willingness to participate in the project by giving a written consent. Before the study was initiated, the parents/legal guardians gave their informed consent to participate in the study of their children. The study was conducted in accordance with the Declaration of Helsinki, and the protocol was approved by the Ethics Committee of the University of Rzeszow/Poland (resolution 2/06/2010).

### Methods

#### Physical fitness assessment

The assessment of the level of physical fitness of children was made using the Wroclaw Physical Fitness Test for preschool children developed by Sekita^29^. The test for preschool children included shuttle run 4 × 5 m with moving the block, standing long jump, throwing 1 kg medicine ball with two hands above the head and 20 m run.

Shuttle run 4 × 5 m with moving the block (agility) was adapted to the age of the children. This exercise is a modification of the agility test of the International Physical Fitness Test (IPFT). The modification concerned reducing the distance from 4 × 10 m to 4 × 5 m. The 5 m long surfaces were marked with two lines on which two blocks were placed at a distance of 20 cm. On the signal “run” the child covered the distance of 5 m four times moving the two blocks from the end line to the start line. Each child had two attempts. The time was measured with an accuracy of 0.1 s. The time for the best attempt, i.e. the fastest performed was taken into account.

During the standing long jump (power) the distance from the starting line to the heel or other part of the body that touched the floor closest to the starting line was measured. The child stood with their feet together in front of the starting line. They were ordered to make the longest leap forward. Each child had two attempts. The length was measured with an accuracy of 1 cm. The longest jump was taken into account.

Throwing 1 kg medicine ball with two hands above the head (strength) was measured in standing position. The beginning of the section was marked with a clearly seen line about 60 cm long. From this point the child made three throws. Then, every 10 cm lines were made on the floor parallel to the initial line indicating the distance in cm. At a distance of about 70 cm for younger children and about 100 cm for older children away from the starting line, chairs were arranged so that the width between them was about 1.5 m. A tape was hung between the backs of the chairs. The children were asked to do the throws over the suspended line. This eliminated the tendency to throw the ball down in front of oneself. The distance to the ball thrown from above the head was assessed. The distance was measured with an accuracy of 10 cm, and the greatest distance was taken into account for each child.

Speed test (20 m run from high start)—a flag was set at the beginning of the line, thus determining the “start” place. At a distance of 20 m from the starting line, a thin parallel line was marked as the time measurement point, and 5 m further a second flag was placed, meaning “finish”. The tested child stood on the line and was instructed as follows: “on the “start” signal you will run as fast as you can to the second flag”. Each child performed the test twice. The test time was measured with an accuracy of 0.1 s. The time for the best test, i.e. the fastest performed was taken into account.

The standing long jump, shuttle run 4 × 5 m with moving the block and throwing 1 kg medicine ball were performed by the children in a closed room, while the speed test (20 m run) was done outside the building. The tests were performed in preschool rooms and areas adjacent to the institution (playgrounds, green area). During testing, efforts were made to maintain equal measurement conditions. Tests were preceded by a short warm-up. Before performing, each test was thoroughly demonstrated by the examiner and each child was given instructions on how to do it. The same measuring instruments were always used during the tests.

#### Statistical analysis

The LMS method was used in the study^30^. Parameters L, M, and S allow estimating the value of any centile for the age. In accordance with the WHO guidelines, while creating percentile charts the percentile values and LMS values were presented with an accuracy of 4 decimal numbers. This ensured appropriate smoothing of obtained centile curves by the age.

## Results

Table 1 presents the results of individual motor tests taking into account the age and sex of the subjects. The analysis showed that boys present a higher level of tested abilities compared to girls. This phenomenon is observed in every age group and in the scope of all tested parameters—agility, power, strength and speed. At the same time, an increase in the level of analyzed motor skills is observed with the growing age of the girls and boys studied.

**Table 1:** Results of individual physical fitness of Polish children aged 4–7 years.

Age/sex	N	Agility (s)		Power (cm)		Strength (cm)		Speed (s)	
		$\bar{x}$	sd	$\bar{x}$	sd	$\bar{x}$	sd	$\bar{x}$	sd
4 years	1925	11.60	1.34	82.83	18.04	175.90	39.63	6.18	0.74
Girls	927	11.71	1.34	80.64	18.14	169.36	37.76	6.27	0.77
Boys	998	11.50	1.33	84.86	17.70	181.97	40.37	6.10	0.70
5 years	4231	10.67	1.26	95.22	17.83	206.88	45.43	5.78	0.76
Girls	2052	10.73	1.24	93.93	17.37	202.37	42.99	5.82	0.76
Boys	2179	10.60	1.28	96.44	18.18	211.12	47.24	5.75	0.76
6 years	4359	9.96	1.17	105.67	17.66	243.51	55.21	5.38	0.68
Girls	2125	10.08	1.11	103.44	16.74	232.90	47.83	5.44	0.68
Boys	2234	9.86	1.22	107.79	18.23	253.60	59.70	5.32	0.68
7 years	1194	9.52	1.02	112.19	17.07	270.29	59.37	5.17	0.57
Girls	580	9.67	1.01	108.86	16.68	257.84	52.95	5.22	0.57
Boys	614	9.38	1.02	115.34	16.85	282.05	62.65	5.13	0.58
Total	11709	10.44	1.38	98.80	19.91	221.89	57.79	5.64	0.78

**Figure 1:**
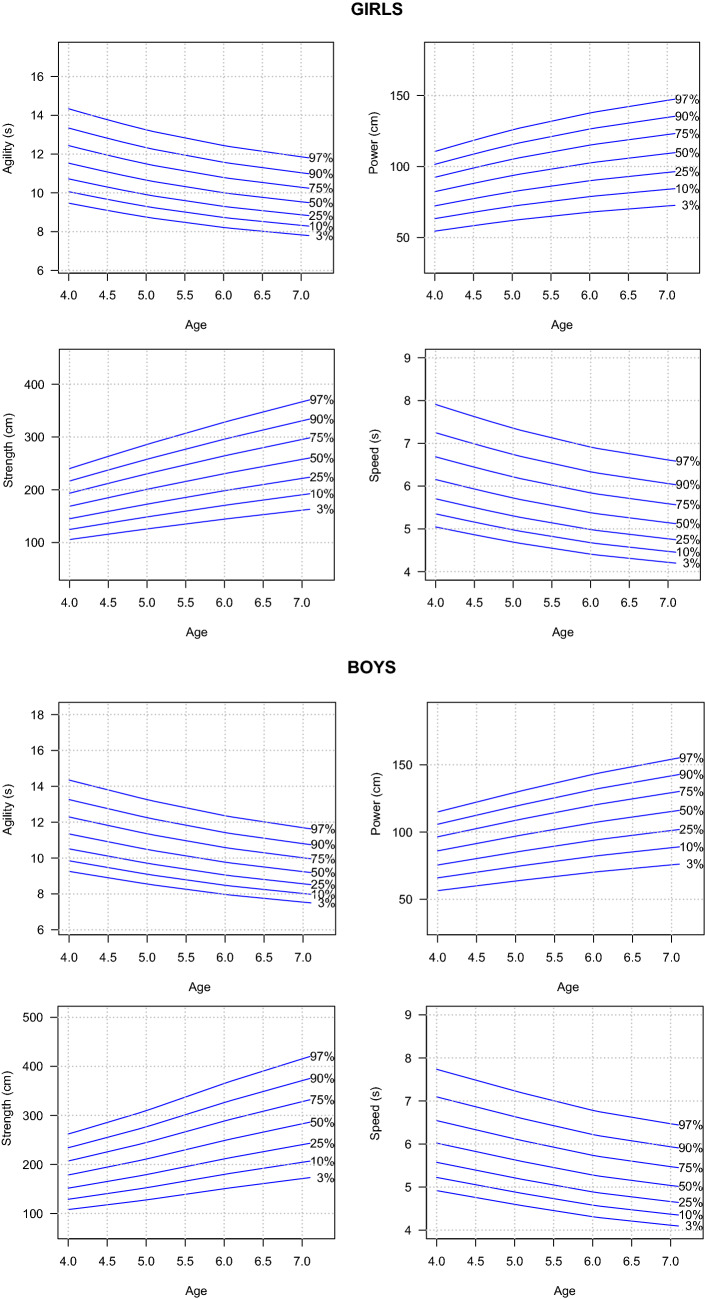
Percentiles charts of physical firness of Polish children aged 4–7 years.

Table 2 presents smoothed percentiles (P3, P10, P25, P50, P75, P90 and P97) for sex and age in terms of physical fitness tests in a group of boys and girls aged 4 to 7. In the case of the tests where the result of the test was to obtain the shortest possible time (shuttle run 4 × 5 m and 20 m run), lower values indicate a higher level of these abilities. Therefore, the percentiles should be interpreted in the opposite way, i.e. P3 is better than P10. For the same tests of physical fitness, smoothed LMS curves for the 3rd, 10th, 25th, 50th, 75th, 90th and 97th percentile are shown in Fig. 1. Our data show a linear improvement in agility, power, strength and speed in both sexes.

**Table 2:** Percentils of physical fitness of Polish children aged 4–7 years.

Age	Girls (N=5684)						
	3%	10%	25%	50%	75%	90%	97%
**Agility (s)**							
4 years	9.47	10.06	10.72	11.53	12.44	13.34	14.34
5 years	8.75	9.30	9.91	10.66	11.49	12.33	13.25
6 years	8.21	8.73	9.30	10.00	10.79	11.58	12.44
7 years	7.83	8.32	8.87	9.54	10.29	11.04	11.86
**Power (cm)**							
4 years	54.53	63.33	72.30	82.33	92.41	101.53	110.57
5 years	62.02	72.03	82.24	93.65	105.12	115.49	125.77
6 years	67.90	78.86	90.03	102.52	115.07	126.43	137.68
7 years	72.28	83.95	95.85	109.14	122.51	134.59	146.57
**Strength (cm)**							
4 years	105.76	124.86	145.17	168.81	193.52	216.63	240.22
5 years	125.85	148.57	172.74	200.87	230.27	257.78	285.85
6 years	144.47	170.55	198.29	230.58	264.34	295.91	328.13
7 years	161.40	190.53	221.52	257.60	295.31	330.57	366.57
**Speed (s)**							
4 years	5.05	5.35	5.70	6.15	6.68	7.25	7.91
5 years	4.69	4.98	5.30	5.72	6.22	6.74	7.35
6 years	4.41	4.68	4.98	5.38	5.84	6.33	6.91
7 years	4.22	4.47	4.77	5.14	5.59	6.06	6.61

Boys (N = 6025)							
**Agility (s)**							
4 years	9.26	9.85	10.51	11.34	12.29	13.26	14.35
5 years	8.55	9.10	9.71	10.48	11.36	12.25	13.26
6 years	7.97	8.48	9.05	9.77	10.58	11.42	12.36
7 years	7.54	8.02	8.56	9.24	10.01	10.80	11.69
**Power (cm)**							
4 years	56.41	65.92	75.47	85.99	96.43	105.77	114.94
5 years	63.57	74.28	85.05	96.90	108.67	119.19	129.53
6 years	70.17	82.00	93.87	106.96	119.95	131.57	142.97
7 years	75.62	88.37	101.17	115.28	129.28	141.79	154.09
**Strength (cm)**							
4 years	108.01	128.98	151.67	178.52	207.06	234.12	262.09
5 years	127.65	152.43	179.24	210.98	244.70	276.69	309.74
6 years	150.47	179.68	211.28	248.70	288.44	326.15	365.11
7 years	171.15	204.38	240.33	282.88	328.09	370.98	415.30
**Speed (s)**							
4 years	4.92	5.22	5.57	6.02	6.54	7.10	7.74
5 years	4.60	4.89	5.21	5.63	6.12	6.64	7.24
6 years	4.31	4.58	4.89	5.28	5.74	6.22	6.78
7 years	4.11	4.37	4.66	5.04	5.47	5.94	6.47

## Discussion

Currently, physical fitness is considered to be an important health indicator^6,7^. However, the diagnostic significance of physical fitness tests for children’s health is often neglected both at schools and at lower levels of education. It is known that the faster abnormalities in motor development of children are detected, the greater the chance of improving their health in adulthood. Many studies emphasize that children with motor disabilities are characterized by a low level of aerobic fitness^31–33^, which can lead to many diseases in adulthood. Considering that individual elements of physical fitness are associated with different health issues, these consequences constitute a concern for the current and later health condition of children with low motor competence^34^. Additionally, the term “developmental coordination disorder” (DCD) appears in the literature. It is a disorder in which the child has a problem, including the whole body movements and coordination, which causes problems with walking, running or jumping^35^. Research by Schott et al. suggests that children with DCD are prone to low levels of physical fitness compared to their peers^31^.

This work is the first attempt to provide physical fitness standards for preschool children based on research conducted for over 3 years (2016-2019). The study used 4 tests of physical fitness—agility, power, strength and speed. The analysis showed that the differences in the level of physical fitness between girls and boys are already visible in early childhood. This phenomenon is confirmed by many authors^36–39^.

Generally, there is an opinion that it is the school period that is the most appropriate stage to identify children with low levels of physical fitness and promote healthy behaviors^6^. In fact, a significant number of children attend kindergarten. For example, in Poland, in 2017/2018, over 1 million children attended this type of institution (data from GUS—head statistics office in Poland). This means that at this stage of education you can already diagnose physical fitness and refer to the norms for a given age. This would allow us to detect the irregularities in their development even earlier. Children who score below P3 should be referred for further diagnosis in order to determine other possible risk factors that may cause a disease^28,40^.Scientists from Germany came to similar conclusions^27^. They developed percentile fitness charts for children aged 9–12. According to them, this type of research is important not only for detecting developmental abnormalities. Percentile charts are an important source of information for professionals—trainers, instructors or physical education teachers. On their basis sports talents can be discovered^27^. Fitness reference standards have also been created for Spanish children aged 6–17. The percentile values presented concerned four aerobic fitness tests. The results of the Spanish population survey confirmed frequent sex differences in the studied parameters and it was noticed that boys perform better than girls^41^. A similar phenomenon was observed in this study—boys show a higher level of physical fitness compared to girls in each age group. This trend was also confirmed among the Portuguese children’s population^26^. Boys performed better than girls in each physical fitness test except for the modified-back-saver-sit-and-reach tests. Other reports show that boys performed better than girls in such activities as catching, throwing and standing long jump^42,43^.

Preschool children are in the phase of continuous motor, physiological and psychological changes^44^. We noticed that older children (both boys and girls) achieved better results in individual physical fitness tests compared to their younger colleagues. A similar phenomenon has been observed in many studies^45,46^.Comparing the results of Polish children with the results of the research conducted among the Portuguese population^45^ it is noted that the Polish population of boys and girls aged 6–7 presents a higher level of strength (standing long jump). For example, the P50 value in standing long jump in the group of Polish boys aged 6 is 106.96 cm. and in the Portuguese population 98.0 cm^45^. This tendency is also observed when comparing own results with the results from Macedonia^47^ and Southern Spain^48^. Correlating the presented parentel values in terms of power to the European data^49^ a higher level of strength (standing long jump) of Polish girls and preschool boys is also found. The P3 value for 6-year-old girls from the European population for the standing long jump is 56.6 cm, and P97—127.0 cm. However, for Polish girls the P3 value is 67.90 cm and P97— 137.68 cm. Comparing the results of own research with the research by Sanchez et al. a similar phenomenon is observed. The population of Polish preschool children shows a much higher level of lower-limb muscular strength (power) compared to the population of Spanish children. We observed that the P50 value in the studies by Sanchez et al. was definitely lower than in our research. This phenomenon was observed in all age groups for both sexes^50^.

Given the differences in the methodology used, we are unable to compare our agility results with other studies. In most of the papers, the authors use the 4 × 10 m shuttle run test to assess agility. A shuttle run 4 × 5 m with moving the block was used in this study. Nevertheless, it was observed that P50 showed the same trend for boys and girls, improving agility with the age of the subjects, and the extent of the difference between the ages was systematic (0.4 to 0.8 s). Analyzing the speed test, we found only one paper in which the authors provide reference values using the 20 m run from high start test. P50 values for the speed test among the Lithuanian population of girls aged 6 and 7 were 5.96 s and 5.20 s, respectively, while for boys of the same age, 5.50 s and 5.02 s, respectively. Comparing the data of the Lithuanian population with the Polish population, it is noted that the results show comparable values, with a slight predominance of the Polish children. Nevertheless, better results in older children can be explained by the development of motor coordination in the preschool period. Improvements in speed scores were also shown among the European child population aged 6 to 9 years^25^.

In terms of fitness, upper body muscle strength is considered to be a health condition predictor^51^. One of the most popular tests to assess upper limb muscle strength is handgrip test^28,45,50,51^. In this study, to assess the fitness of the upper limbs , it was decided to throw a medicine ball, which does not require special tools to evaluate this parameter. To our knowledge, this is the first study to provide a reference value for throwing a 1 kg medicine ball with two hands above the head in a large sample of preschool children. For example, Vaccari et al. also developed their own test to check the strength of the muscles of the upper limbs in the population of Italian children aged 6–11 years^52^. The results of their research indicate an increase in the level of the tested ability with the age of the respondents (both in boys and girls). Additionally, it was observed that girls are characterized by lower efficiency of the upper limbs compared to boys.This phenomenon is also observed in these studies.

From a practical point of view, the developed reference standards of physical fitness allow us to classify preschool children in terms of the level of physical fitness: very poor (X <P10); poor (P10 ≤ X <P25); medium (P25 ≤ X < P75); good (P75 ≤ X < P90); and very good (X < P90). According to Miguel-Etayo et al.^25^ physical fitness below P5 may be a potentially pathological condition and a warning sign. Poor physical fitness outcomes may prompt the directly responsible persons, such as parents, doctors or school community, to solve the problem. Additionally, the authors conclude that children below P25 could be included in a physical activity program to improve their fitness, and children with higher percentile values (e.g. P90) could be directed towards further sports development.

Research limitations relate to the lack of basic anthropometric measurements (height and weight). Additionally, there are differences in biological age already at the preschool age. This means that more biologically mature children have a higher level of physical fitness. In some cases, the results may not be reliable, as the biological age can differ from the calendar age. The strength of the study is the large population of tested children aged 4-7. The use of GAMLSS as a powerful tool to obtain smooth age-dependent reference curves is also a significant advantage of the study.

## Conclusions

Based on the conducted research, percentile charts for four physical fitness tests of preschool children were developed. The presented parentel values can be helpful to classify and estimate the percentage of children with low or high levels of physical fitness. These findings will help health, sports, school and kindergarten workers. Our research shows that sexual dimorphism is already seen at the age of 4. In general, boys present a higher level of physical fitness than girls.
